# Accuracy of Surface Electromyography in the Diagnosis of Pain-Related Temporomandibular Disorders in Children with Awake Bruxism

**DOI:** 10.3390/jcm11051323

**Published:** 2022-02-28

**Authors:** Liliana Szyszka-Sommerfeld, Magdalena Sycińska-Dziarnowska, Agata Budzyńska, Krzysztof Woźniak

**Affiliations:** Department of Orthodontics, Pomeranian Medical University in Szczecin, Al. Powst. Wlkp. 72, 70-111 Szczecin, Poland; magdadziarnowska@gmail.com (M.S.-D.); klimaga@op.pl (A.B.); krzysztof.wozniak@pum.edu.pl (K.W.)

**Keywords:** awake bruxism, bruxism, muscle activity, myofascial pain, orofacial pain, pain-related temporomandibular disorders, surface electromyography, temporomandibular disorders

## Abstract

The study assessed masticatory muscle electromyographic (EMG) activity in both children diagnosed with pain-related temporomandibular disorders (TMD-P) and awake bruxism (AB) and in children without TMD, as well as the diagnostic value of surface electromyography (sEMG) in diagnosing TMD-P in subjects with AB. After evaluation based on the Axis I of the Research Diagnostic Criteria for Temporomandibular Disorders (RDC/TMD), 30 children diagnosed with myofascial pain were included in the myofascial pain group and 30 children without TMD diagnosis comprised the control group (mean age of 9.49 ± 1.34 years). The activity of the anterior temporal (TA) and masseter (MM) muscle was assessed bilaterally using a DAB-Bluetooth device (zebris Medical GmBH, Germany) at rest and during maximum voluntary clenching (MVC). The receiver operating characteristic (ROC) curve was used to determine the accuracy, sensitivity, and specificity of the normalized sEMG data. Statistically significant intergroup differences were observed in TA and MM muscle EMG activity at rest and during MVC. Moderate degree of sEMG accuracy in discriminating between TMD-P and non-TMD children was observed for TA_mean_, left MM, and MM_mean_ EMG muscle activity at rest. sEMG can be a useful tool in assessing myofascial TMD pain in patients with AB.

## 1. Introduction

Bruxism is an umbrella term for various masticatory muscle motor phenomena/behaviours of the masticatory muscles [1]. According to the current international consensus on the assessment of bruxism, awake bruxism (AB) is defined as “masticatory muscle activity during wakefulness that is characterized by repetitive or sustained tooth contact and/or by bracing or thrusting of the mandible” [2]. Furthermore, AB “is not a movement disorder in otherwise healthy individuals” according to the current consensus but rather a behaviour that may be a risk (and/or protective) factor with some possible clinical consequences. In this light, bruxism is seen as a risk factor that may have possible negative oral health results, such as severe pain in the masticatory muscles or temporomandibular joint (TMJ), extreme mechanical tooth wear, cracked teeth, and/or prosthodontic problems [2,3]. Consequently, people with AB are more likely to develop pain-related temporomandibular disorders (TMD-P) [1,3,4,5] due to overload of musculoskeletal structures and craniofacial pain [6,7], although the relationship between pain and bruxism remains unclear [8,9,10].

The main symptom of TMD-P is persistent, recurrent, or chronic pain that affects the masticatory muscles, TMJ, and/or adjacent structures [11,12]. The prevalence of temporomandibular disorders (TMD) in children and adolescents ranges from 9.8% to 80% and for TMD-P from 1% to 22% [7,11]. The aetiology of TMD-P is believed to be multifactorial and the result of a complex interaction between biological, psychological, social, and environmental variables [12,13]. The multifactorial aetiology of this condition makes it difficult to make an accurate and precise diagnosis and appropriate tools and measures are needed for proper assessment [14,15,16,17]. One of the most current and reliable tools for assessing TMD in both children and adults is the Research Diagnostic Criteria for Temporomandibular Disorders (RDC/TMD), which helps identify TMD by diagnosing physical aspects, pain-related disability, and psychosocial factors [18,19]. However, the limited cognitive ability of many children, especially the youngest, to answer the questionnaire and to undergo a physical examination may undermine the reliability of the results. Nevertheless, it is the validated diagnostic method in young patients. Although the Diagnostic Criteria for Temporomandibular Disorders (DC/TMD) was recently published as RDC/TMD adaptation, it has not yet been validated for children [9]. In this light, certain instruments may provide important quantitative data that may prove useful in the clinical assessment of TMD [16,17].

Bruxism is more common in children than in adults and less prevalent in the elderly as the importance of this condition decreases with age. The prevalence of bruxism in children ranges from 3.5% to 40.6%, and there is no gender preference [9,20]. Barbosa et al. [10] found that the prevalence of sleep bruxism (SB) in childhood and adolescence ranges between 7.0% and 15.1%, with girls being more frequently affected. The aetiology of bruxism results from a complex interaction between peripheral (morphological) and central (pathophysiological and psychological) variables [21,22,23]. It has also been reported that in younger children, the immaturity of the masticatory neuromuscular system may play a role [10].

Both non-instrumental (especially self-reporting) and instrumental (notably electromyography-EMG) methods can be used in evaluation of bruxism, leading to three degrees of probability of correct diagnosis AB and SB, namely “possible” (positive self-report only), “probable” (positive clinical evaluation with or without positive self-report) and “definite” (positive instrumental assessment with or without positive self-report and/or positive clinical inspection) [2]. Instrumental methods, such as surface electromyography (sEMG) during wakefulness, can provide evidence of AB, although there are no protocol recommendations yet [1,24]. In a review of the literature, Yamaguchi et al. [25] revealed that to assess AB, EMG measurements of masticatory muscle activity can be easily and accurately performed during the day using wearable EMG devices.

sEMG is a widely used non-invasive technique to identify electrical signals on the skin above superficial muscles [26]. sEMG signals, conducted through tissues and detected by surface electrodes, show the temporal and spatial summation of populations of adjacent motor units [27]. This method has found application in the diagnosis of patients with general muscle disorders, neuromuscular diseases, or diseases affecting neuromuscular performance [28]. sEMG has several applications in dentistry, including orthodontics, implants, occlusion, bite correction, TMJ disorders, and sleep disorders [29]. The advantages of this instrument-based method are its non-invasiveness, simplicity, and accessibility [26]. However, it also has a number of disadvantages. Firstly, any analysis of sEMG recordings is limited to assessing overall muscle activity, cooperation between different muscles, and variability in their activity over time by detecting with surface electrodes the superimposed motor unit action potentials from numerous muscle fibers. Another disadvantage of sEMG is its sensitivity to impedance imbalance, which may reduce the accuracy and reproducibility of EMG assessment. This limitation can be overcome by establishing a constant distance between the electrodes, developing a standard procedure for surface electrode placement and performing an adequate quantitative analysis of the sEMG recordings based on normalization procedures [26,28].

The use of sEMG as a tool to assess individuals with TMD, including pain-related TMD is still debated due to the significant variability in results reported in the literature. The diagnostic value of sEMG in the evaluation of this condition is not yet clearly established [17,28,30,31]. There is also little information on the electromyographic analysis of masticatory muscle activity in children diagnosed with TMD-P and further studies are needed in this area, especially in young patients [11,32,33]. Accurate diagnosis of TMD-P in children is particularly important because children usually have difficulty expressing unpleasant pain symptoms, so they may be overlooked. To date, there have been no studies on the reliability and validity of sEMGs in the diagnosis of myogenous TMD pain in children with AB.

The first aim of this study was to assess the EMG activity of the masticatory muscles both in children diagnosed with myofascial TMD pain and AB and in children without TMD. Another objective was to evaluate the sensitivity, specificity, and accuracy of sEMG in the diagnosis of pain-related TMD in children with AB. The null hypothesis is that no differences exist between children with myofascial pain and AB and asymptomatic children when it comes to the EMG activity of the masticatory muscles at rest and during maximum voluntary contraction (MVC).

## 2. Materials and Methods

The study protocol was in accordance with the guidelines of the Helsinki Declaration and approved by the Local Bioethics Committee of the Pomeranian Medical University (project number KB-0012/08/15). After obtaining information about the study project, parents/guardians gave written consent for their children’s voluntary participation in the planned procedures.

One hundred and thirty children aged 8–11 years who met the following inclusion criteria were consecutively recruited for the study: the presence of mixed dentition, male and female gender, no history of trauma or orthodontic treatment, and consent to participate voluntarily in the study. All patients in the initial sample underwent a thorough examination carried out by the same trained and experienced examiner, including the assessment of the presence of TMD using Axis I of the RDC/TMD, bruxism, and occlusal characteristics. Of the 130 initially screened participants who were referred to the Orthodontics Outpatient Clinic in Szczecin, Poland, in January 2020, 60 children were included in the final data analysis after applying the exclusion criteria and matching for occlusal characteristics. The final study sample comprised of 60 children of both sexes with a mean age of 9.49 ± 1.34 years. After evaluation based on the algorithms for Axis I of the RDC/TMD [18], 30 children with a diagnosis of myofascial pain (Group Ia and Ib) were included in the myofascial pain group (mean age of 9.65 ± 1.25 years) while 30 children without TMD diagnosis comprised the control group (mean age of 9.33 ± 1.42 years). The study groups were matched for occlusal characteristics. All subjects in the myofascial pain group were diagnosed with AB. The presence of bruxism was diagnosed according to the criteria of the American Academy of Sleep Medicine (AASM) [34] and was based on (1) self-reporting of bruxism, and (2) a clinical examination to evaluate bruxism signs. Inclusion criteria for the diagnosis of AB were consistent with the international consensus for the assessment of bruxism presented by Lobbezoo et al. [2]. The control group included children without clinical signs and symptoms of AB.

Subjects meeting the criteria for a diagnosis of Axis I of the RDC/TMD without pain or with a diagnosis of TMJ pain, subjects with a history of cervical spine or TMJ surgery and trauma, a history of neuromuscular illness or disease affecting neuromuscular performance, subjects with the presence of SB, dental caries, early tooth loss, systemic, rheumatologic and/or mental disorders, asthma/other respiratory disease as well as subjects taking medications that could affect muscle function were excluded from the study. Subjects diagnosed with myofascial pain without AB as well as subjects with a diagnosis of AB without myofascial pain were also excluded.

In the first part of the examination, the general medical records of the patients and questionnaires conducted with the parents/guardians were analysed. General medical records included demographic and social data, as well as information about a history of surgery and trauma, and systemic medical conditions or medications that could affect muscle function. A questionnaire interview was designed to obtain information about oral history, masticatory muscle function, oral parafunctional habits, jaw/teeth clenching and bracing/thrusting, and teeth touching during non-swallowing behaviour, as well as questions about subjective TMD symptoms (jaw pain during function, headaches, jaw stiffness/fatigue, difficulty in opening the mouth wide, teeth grinding, and TMJ sounds/pain). For the self-reporting of bruxism, the following two questions modified from van der Meulen et al. [35] were asked: (a) do you grind your teeth or do you clench your jaws during wakefulness? and (b) did someone notice or are you yourself aware that you grind your teeth or clench your jaws during sleep? Parents, in collaboration with their children, completed the questionnaire and they could only answer yes/no/I don’t know; parents were instructed to answer ‘yes’ to the questions asked if they considered their children’s habit was regular or frequent, but additional questions regarding frequency and timeframe were not asked [3,21,36,37].

Body mass index (BMI) percentiles for age were calculated for all children using the BMI-for-age growth chart. The children were then examined by the same experienced examiner for the presence of TMD. The clinical signs of TMD were assessed using Axis I of the RDC/TMD, including pain on palpation, mandibular range of motion (mm), associated pain (jaw opening pattern, unassisted opening, maximum assisted opening, mandibular excursive and protrusive movements), TMJ sounds, and tenderness induced by muscle and joint palpation [18,38]. The myofascial pain group based on the self-report, clinical criteria, and diagnosis included subjects with myofascial pain Ia, Ib (pain of muscular origin, including pain experienced in the face or masticatory muscles at rest or during function, and pain on palpation at three or more sites). During the clinical examination in children, the following clinical signs of bruxism were also evaluated: (a) abnormal tooth wear, (b) teeth impressions in the buccal region, (c) teeth impressions on the tongue, and (d) masticatory muscle pain, or muscle fatigue and hypertrophy [2,34,39,40].

An analysis of the dental arch shape in three planes was performed along with a reciprocal analysis of both dental arches to assess occlusal characteristics, such as the sagittal relationship of the permanent first molar according to Angle’s classification, posterior crossbite, overbite, overjet, and lateral open bite.

Intraclass correlation coefficients (ICCs) were used to evaluate intra-examiner reproducibility in clinical diagnoses of TMD and AB in 20 randomly selected children. The considered ICC values were as follows: ICC < 0.4, corresponding to poor reliability; 0.4 ≤ ICC ≤ 0.75—fair to good reliability; ICC > 0.75—excellent reliability [41,42].

The activity of both masseter and anterior temporal muscles was recorded with DAB-Bluetooth electromyography (zebris Medical GmbH, Isny im Allgäu, Germany) by an experienced researcher who was blinded for the myofascial pain and control groups. Four channels of the device were used with simultaneous acquisition for the EMG recording, common grounding for all channels, a high-pass filter of 7 Hz–5 kHz, 12 bits of dynamic resolution range, a channel sampling frequency of 1 kHz, and an input impedance of 146 kΩ for the analogue channels.

Four disposable Ag/AgCl bipolar surface electrodes (Noraxon Dual Electrode, Noraxon, Scottsdale, AZ, USA) were placed bilaterally at a constant inter-electrode distance of 20 mm on the anterior portion of the right (RTA) and left (LTA) temporal muscles—vertically along the anterior margin of the muscle and approximately over the coronal suture, as well as on the superficial part of the right (RMM) and left (LMM) masseter muscles—parallel to the muscular fibers, with the upper pole of the electrode located at the intersection between the tragus-labial commissura and the exocanthion-gonion lines [43]. During the measurement of muscle EMG activity, the subjects were comfortably seated in an upright position in a dental chair with the head in its natural head position [44]. The reference electrode was attached below and behind the right ear.

Before the sEMG measurements, the patient’s skin was degreased with 70% ethyl alcohol solution at the electrodes contact areas to reduce impedance. Proper preparation of the investigated area after placement of the surface electrodes was monitored by an impedance test using a Metex P-10 measuring device (Metex Instruments Corporation, Seoul, Korea). The skin impedance had to meet the target parameters of 1 × 10^3^–30 × 10^3^ Ω before the start of sEMG recordings.

The sEMG measuring procedure commenced 5 min later. Prior to the EMG examinations, patients underwent a brief training to familiarize them with the tasks. The activity of the anterior temporal (TA) and masseter (MM) muscles was evaluated by sEMG recordings under the following clinical conditions: (1) in the mandibular rest position; (2) during maximum voluntary clenching (MVC) when children were asked to keep their teeth clenched in an intercuspal position as hard as possible for a duration of 5 s; (3) during MVC with two 10-mm cotton rolls bilaterally placed on the occlusal surfaces of the posterior teeth when subjects were asked to keep their teeth clenched as hard as possible for 5 s. The tasks were repeated three times, and the sEMG data used in the procedure constituted the arithmetic means of the last two repetitions [45,46].

The sEMG signals were filtered, amplified and digitized. The sEMG data were then recorded by computer and normalized to ensure a reliable further analysis. The normalization process was the primary component of the data analysis. The clenching test with two cotton rolls (standardization test) provided reference EMG values for the normalization procedure. For each of the analysed muscles, the mean EMG potentials during MVC using cotton rolls were set at 100%. Data normalization was therefore performed according to the following formula: mean EMG values at rest or during MVC (µV)/mean EMG values during MVC with cotton rolls (µV) × 100% (μV/μV%). This procedure was necessary for the preliminary processing of raw data to ensure intercomparisons analysis. According to this protocol, normalized EMG data will provide information on the impact of occlusion on neuromuscular activity, while avoiding individual variability (anatomical variations, physiological and psychological status, etc.) and technical variations (muscle cross-talk, electrode position, skin and electrode impedance, etc.) [47,48,49].

The Asymmetry Index (AsI, range from 0% to 100%), assessing asymmetry between the EMG activity for the left and right masticatory muscles, was calculated according to the following equation [50]:

AsI = ∑i = 1N|Ri − Li|/∑i = 1N(Ri + Li) × 100

Reproducibility of the sEMG procedure was tested using duplicate sEMG recordings in 20 subjects performed by the same examinator. Between two sEMG measurements, children were asked to relax for 15 min.

The statistical analyses were performed using the STATISTICA 13.0 PL for Windows software package (StatSoft Poland, Cracow, Poland). The Levene test was used to evaluate homogeneity of variance. Normality was assessed with the Kolmogorov–Smirnov test. To verify the study hypothesis regarding the presence or absence of differences in masticatory muscle EMG activity between the myofascial pain and non-TMD groups, the Student *t* test and the Mann-Whitney U test were applied. The chi-square test was used to determine differences between the groups with regard to the prevalence of analysed variables, such as gender, BMI, and occlusal characteristics. The level of significance was set at *p* = 0.05. Statistical differentiation of myofascial pain AB cases compared with the control group without TMD required the use of a receiver operating characteristic (ROC) curve. Therefore, the cut-off values of the continuous variables for the myofascial pain AB group relative to the control group were determined by means of ROC curves analysis. The results were described by the area under the curve (AUC), the standard error of AUC score (SE), 95% Confidence Interval for AUC, the *p* value and the ROC coordinate curves. The sensitivity (Se) and specificity (Sp) of the test group relative to the reference group were estimated. The AUC was classified as follows: “0.5—result due to chance; >0.5 to 0.7—low accuracy; >0.7 to 0.9—moderate accuracy; >0.9 to <1.0—high accuracy; and 1.0—a perfect test” [51].

## 3. Results

The reliability of the clinical diagnoses of TMD-P and AB in children ranged from good to excellent.

Table 1 presents the characteristics of the study groups. The groups were similar with regard to age, gender, BMI, and occlusal characteristics (*p* > 0.05).

No significant differences (*p* > 0.05) were observed between duplicated sEMG recordings in all analysed variables.

The electromyographic activity of the TA and MM muscles at rest and during MVC for both the myofascial pain and control groups is presented in Table 2 and Table 3. Analysis of the sEMG recordings revealed statistically significant intergroup differences in the EMG activity of the temporal and masseter muscles at rest and during MVC. The EMG potentials of the right and left masticatory muscles at rest were significantly higher in the myofascial pain group compared to the control group (Table 2). During MVC, temporal (RTA) and masseter (LMM, MM_mean_) muscle EMG activity was significantly lower in children with TMD-P and AB than in those without TMD (Table 3). No significant intergroup differences were observed in the Asymmetry Index for the TA and MM muscles at rest and during MVC (Table 2 and Table 3). There were no significant differences between females and males in each group regarding TA and MM muscle EMG activity and the Asymmetry Index at rest position and during MVC (Table 2 and Table 3).

Table 4 and Table 5 show the diagnostic efficiency of sEMG in identifying subjects with pain-related TMD. The analysis of the ROC curve showed that the highest diagnostic efficiency of sEMG in discriminating between myofascial pain and non-TMD subjects was observed in the case of estimators of the distribution of variables, such as temporal (RTA, TA_mean_) and masseter (LMM, RMM, MM_mean_) muscle activity in a rest position (Table 4), as well as RTA and MM_mean_ muscle activity during MVC (Table 5).

A moderate degree of sEMG accuracy in terms of differentiating between myofascial pain and non-TMD children according to the AUC classification was observed for TA_mean_ (AUC = 0.738, Se = 66.7%, Sp = 66.7%, cut-off point value = 5.75 μV/μV%), LMM (AUC = 0.711, Se = 76.7%, Sp = 60.0%, cut-off point value = 3.80 μV/μV%) and MM_mean_ (AUC = 0.744, Se = 86.7%, Sp = 60.0%, cut-off point value = 3.45 μV/μV%) muscle EMG activity in a rest position (Table 4).

## 4. Discussion

This study evaluates the electromyographic activity of the masticatory muscles in children with clinically diagnosed myofascial TMD pain and AB. Furthermore, the present study provides data on the diagnostic efficiency of sEMG in the assessment of pain-related TMD in children with AB. The sEMG recordings showed statistically significant intergroup differences in masseter and anterior temporal muscle EMG activity at rest and during MVC. The analysis of the ROC curve demonstrated that the highest diagnostic efficiency of sEMG in discriminating between myofascial pain and non-TMD subjects was observed for temporal (RTA, TA_mean_) and masseter (LMM, RMM, MM_mean_) muscle activity at rest, as well as for RTA and MM_mean_ muscle activity during MVC. The accuracy of sEMG in differentiating between children with and without TMD was moderate in the case of TA_mean_, LMM, and MM_mean_ muscle EMG activity at rest.

The present study comprised subjects matched for morphologic occlusion, as this is a factor that may affect the electromyographic activity of the masticatory muscles. The myofascial pain group included only muscle-related pain disorders according to the RDC/TMD Axis I diagnostic criteria. In this way, we ensured sample homogeneity and reduced the number of confounding factors. We compared the electrical potentials of the masticatory muscles using sEMG in children diagnosed with myogenous TMD pain and AB with those of children without signs and symptoms of TMD and bruxism. Muscle activity was analysed in two functions: in the mandibular rest position and during MVC in the intercuspal position. Normalized sEMG data were used because the normalization process is essential for the initial processing of raw data to ensure interindividual comparability. The study revealed higher temporal and masseter EMG muscle activity at rest and lower EMG potentials of the temporal (RTA) and masseter (LMM, MM_mean_) muscles during MVC in children diagnosed with myofascial pain and AB than in children without TMD and bruxism. However, when interpreting these study results, it is important to keep in mind that many variables may affect the electromyographic activity of the masticatory muscles e.g., morphologic occlusion, body fat or skull shape. In our study, the groups were similar with regard to age, gender, BMI, and occlusal characteristics were found. Since our study groups included both children with malocclusion and normal occlusion diagnosed on the basis of a thorough clinical examination, cephalometric analysis was not performed in all cases. This was clinically significant in the context of the ALARA (as low as reasonably achievable), the radiology principle of keeping radiation doses delivered to patients as low as reasonably achievable, especially for the youngest patients.

As mentioned earlier, bruxism may be associated with pain and other signs and symptoms in the jaw and face region [52]. Subjects with bruxism are more likely than healthy individuals to keep their teeth in contact and may present with symptoms throughout the day, such as headaches, earache, or muscle soreness due to tension produced during multiple muscle contractions [53,54]. This behaviour can lead to TMD pain due to overload of the musculoskeletal structures and craniofacial pain [6]. Muscle overload due to tooth clenching may be associated with local blood flow and microcirculation disorders, and pain derived from an ischemia [9,55]. Lavigne and Palla [56] suggested that because most SB episodes are phasic, with few tonic or sustained contractions, the probability of reported pain is low when compared to clenching while awake, which is characterized by more sustained contractions. In this context, when interpreting the study results, it is important to keep in mind that the presence of pain in patients with pain-related TMD can reduce the EMG potentials of the masticatory muscles during MVC, while at rest this activity is higher than in asymptomatic subjects [57,58]. The fact that masticatory muscle EMG activity is higher at rest may be due to sensorial–motor interactions, among which pain can modify the formation of action potentials and, finally, muscle electrical activity [59]. In addition, reduced masticatory muscle EMG activity in the myofascial pain group during MVC suggests that there is an alteration in muscle recruitment compared to subjects without TMD. These alterations may be used as an effective protective mechanism for damaged TMJs. Pain induces adaptations by remodeling muscle activity to protect the masticatory motor system from possible trauma. It was indicated that the specific recruitment of the masticatory muscles is due to descending central modulation subsequent to nociceptive stimuli of the affected TMJ and/or myofascial and/or periodontal nociceptors [11,60,61,62]. It should also be noted that the association between pain and muscle activity is still discussed [63].

Only a few masticatory muscle EMG activity studies have been conducted on children with pain-related TMD [11,33], probably because the ability of young subjects to report pain symptoms is difficult and questionable. This is the first investigation on masticatory muscle activity in children diagnosed with AB and myofascial pain based on the RDC/TMD Axis I diagnostic criteria. As no similar studies have been conducted, it is difficult to compare our results with others. Nevertheless, our findings could be set alongside a study by Szyszka-Sommerfeld et al. [11]. The latter authors assessed the EMG activity of the masseter and anterior temporal muscles in 63 cleft lip and palate children aged between 6.4 and 13.9 years: 31 children with TMD-P (both myogenous and arthrogenous TMD according to RDC/TMD Axis I) and 32 children with no TMD diagnosis. Similarly, they found that subjects diagnosed with pain-related TMD have higher temporal and masseter muscle activity at rest and reduced temporal muscle EMG potentials during MVC. Another study [33] assessed the electrical activity of the masticatory muscles in 90 patients aged between 7.1 and 12.6 years divided by RDC/TMD into three groups: a TMD-pain group (included both joint- and muscle-related pain disorders), a pain-free TMD group, and a group without TMD. The authors found that temporal muscle EMG activity at rest in children diagnosed with pain-related TMD was significantly greater compared with the pain-free TMD and non-TMD groups while temporal muscle EMG potentials when clenching were much lower. Moreover, masseter muscle activity at rest in pain-related TMD subjects was significantly higher and masseter muscle electrical potentials during MVC were significantly lower than in patients with no TMD diagnosis. On the other hand, numerous analyses have been conducted on the electrical activity of the masticatory muscles in adults with TMD and TMD-P [17,58,62,64,65,66]. Many authors found that the EMG potentials of the masticatory muscles at rest were higher in a TMD group than in a group without TMD [17,58,62,65,66] and have also reported significantly lower levels of EMG activity during MVC in patients with TMD [17,64,65].

According to the literature, the prevalence of TMD in children is mainly assessed by self-reported or proxy-reported signs and symptoms [67,68]. Therefore, there is a need to develop a more comprehensive standardized process for the diagnosis of TMD in children and adolescents, so that reliability and validity for this population can be assessed and improved [69]. Some authors [19,70] recommend the use of validated tools such as the RDC/TMD in the diagnoses for children and adolescents. However, there are difficulties related to how children express their symptoms and how they respond to physical examination, which may compromise the reliability of the results [9]. Therefore, it is important for practitioners to use complementary assessment tools, including sEMG. The present study assessed the accuracy, sensitivity, and specificity of sEMG in the diagnosis of myogenous TMD pain in children with AB compared with RDC/TMD. Our study confirmed a moderate degree of sEMG accuracy in identifying patients with myofascial pain for such variables as TA_mean_, LMM and MM_mean_ EMG muscle activity at rest (AUC = 0.711–0.744, Se = 66.7–86.7%, Sp = 60.0–66.7%, cut-off values = 3.80–5.75 μV/μV%). These findings may indicate that sEMG is a useful tool in assessing pain-related TMD in children with AB.

The diagnostic efficiency of sEMG in identifying patients with myofascial pain compared with RDC/TMD as the gold standard has not yet been well established. Some studies have demonstrated the accuracy of bioelectric devices in diagnosing myogenous TMD pain compared to RDC/TMD; however, their results are inconsistent [17,28,30,31,65]. In addition, variability in the methodology and study group selection criteria makes it difficult to compare our findings with other study results. Szyszka-Sommerfeld et al. [28] reported a moderate degree of sEMG accuracy based on normalized sEMG data in differentiating between pain-related TMD (both myogenous and arthrogenous) and children without TMD in terms of the mean values of masseter muscle activity and the Asymmetry Index of the masseter muscles at rest (AUC = 0.723–0.728, Se = 87–90%, Sp = 50%, cut-off values = 3.80 μV/μV% and 10.12%). The highest diagnostic efficiency of sEMG in terms of identifying subjects with TMD and TMD-P was observed for the mean values of temporal and masseter muscle EMG activity as well as the Asymmetry Index of the masseter muscles in a rest position. The authors concluded that sEMG could be used as an adjunctive tool in the identification of patients with pain-related TMD. On the other hand, Berni et al. [17], Glaros et al. [66], and Santana-Mora et al. [30] confirmed the diagnostic usefulness of sEMG based on raw sEMG data in the recognition of myogenous TMD in adults. Berni et al. [17] reported a moderate degree of sEMG accuracy in the diagnosis of myogenous TMD in women when it came to anterior temporal, masseter, and suprahyoid EMG muscle activity at rest and suprahyoid muscle activity during MVC on parafilm (AUC = 0.744–0.848, Se = 71.3–80.0%, Sp = 60.5–76.6%, cut-off values = 3.11–9.33 μV). However, they demonstrated the insufficient accuracy of sEMG in the case of masseter and temporal muscle activity during MVC with parafilm (AUC < 0.5). Glaros et al. [66] observed the diagnostic efficiency of sEMG in differentiating between patients with myofascial pain and non-pain control subjects, specifically in the case of left anterior temporal and left masseter muscles (Se = 68.5%, Sp = 66.8%). Santana-Mora et al. [30] revealed the moderate efficiency of sEMG in discriminating between patients with TMD and non-TMD subjects, although only for left temporal muscle activity at rest (AUC = 0.660, Se = 0.547, Sp = 0.842, cut-off point interval = 3.03–22.57 μV). They strongly recommend the application of the indexes (mainly assessing the dominance of temporal over masseter muscles during rest) to increase the discriminatory capacity of raw sEMG evaluation. On the contrary, Manfredini et al. [31] reported insufficient accuracy of sEMG at rest when diagnosing subjects with myofascial TMD pain (AUC = 0.28–0.48) and a moderate degree of sEMG accuracy during clenching tasks (AUC > 0.7). As a consequence, they suggested that instruments such as electromyography might not be useful diagnostic tools for identifying myofascial pain in the masticatory muscles of patients. In light of the above, further research should be conducted to determine the diagnostic usefulness of sEMG in the assessment of pain-related TMD.

### Limitations of the Study

Our study has a number of limitations. The main limitation of our study is the relatively small number of subjects. Further studies involving a larger number of patients are needed to investigate relationship between TMD-P, bruxism, and masticatory muscle activity, as well as the usefulness of instrumental devices in assessing pain-related TMD in young individuals with bruxism.

Another limitation of the study was that the self-reporting of bruxism was based on a questionnaire that only included information regarding the presence of symptoms of bruxism, with no additional questions regarding its frequency and timeframe. This may be significant because self-reporting based only on the prevalence of symptoms of bruxism may lead to an overestimation of the number of patients with bruxism [39,71,72]. Instrument-based studies have shown that people who reported the occurrence of SB on the questionnaires more than once a week are more likely to have moderate-to-high frequency SB on polysomnography than low frequency SB [73,74,75]. In this context, questions about bruxism frequency and timeframe should be considered in future research.

Moreover, multivariate analyses were not performed to control and measure for possible confounders. Further studies are needed that take into account the identification and measurement of multiple potential confounding factors using multivariate analysis.

It should also be noted that our study assessed the EMG potentials of the masticatory muscles in subjects with bruxism and pain-related TMD. As previously mentioned, pain can affect the electrical activity of muscles [59]. Pain is known to lead to adaptations, with reduced muscle activity and restricted movement to protect the system from possible injury [63,76]. The occurrence of pain during muscle contraction can generate greater variability in EMG potentials, which compromises the evaluation of the accuracy of sEMG [17]. In light of the above, in future studies, we also suggest assessing the EMG activity of the masticatory muscles in individuals with bruxism and no pain.

## 5. Conclusions

Based on the results and limitations of the study, the following conclusions can be drawn: children diagnosed with myofascial TMD pain and awake bruxism had altered temporal and masseter muscle EMG activity at rest and during MVC, compared to children without TMD. A moderate degree of sEMG accuracy in terms of discriminating between myofascial pain and non-TMD children was observed for TA_mean_, LMM, and MM_mean_ muscle EMG activity at rest. sEMG can be a useful tool in assessing pain-related TMD in patients with AB. Further studies that include a larger number of patients and take into account various confounding factors are needed to confirm the results of the study.

## Figures and Tables

**Table 1:** The characteristics of the study participants.

Variable		Myofascial Pain Group Mean Age 9.65 ± 1.25	Control Group Mean Age 9.33 ± 1.25	*p*-Value
		*n* (%)	*n* (%)	
Gender	Girls	17 (56.7)	14 (46.7)	0.3777
	Boys	13 (43.3)	16 (53.3)	0.4615
BMI for age	Underweight (<5th percentile)	2 (6.7)	2 (6.7)	1.0000
	Healthy Weight (>5th and <85th percentile)	25 (83.3)	25 (83.3)	1.0000
	Overweight (>85th and <95th percentile)	2 (6.7)	3 (10.0)	0.6404
	Obesity (≥95th percentile)	1 (3.3)	1 (3.3)	1.0000
Vertical overlap	≥0 and <3 mm	17 (56.7)	19 (63.3)	0.5982
	≥3 mm	10 (33.3)	9 (30.0)	0.7814
	Reverse (anterior open bite)	3 (10.0)	2 (6.7)	0.6404
Overjet	≥0 and <3 mm	15 (50.0)	17 (56.7)	0.6048
	≥3 mm	12 (40.0)	10 (33.3)	0.5921
	Negative (anterior crossbite)	3 (10.0)	3 (10.0)	1.0000
Angle Class	I	18 (60.0)	20 (66.6)	0.5921
	II	10 (33.3)	8 (26.7)	0.5731
	III	2 (6.7)	2 (6.7)	1.0000
Posterior crossbite	No	20 (66.7)	22 (73.3)	0.5807
	Yes	10 (33.3)	8 (26.7)	0.5731
Lateral open bite	No	27 (90.0)	28 (93.3)	0.6472
	Yes	3 (10.0)	2 (6.7)	0.6404

**Table 2:** EMG activity of the masticatory muscles at rest in the myofascial pain and control groups.

Region	Variable	Gender	Myofascial Pain Group			Control Group			*p*-Value
			*n*	Mean	SD	*n*	Mean	SD	
RTA	EA	Females	17	5.89	1.65	14	4.88	1.44	0.1877 ^a^
		Males	13	6.75	1.85	16	4.84	2.20	0.9601 ^b^
		Total	30	6.26	1.76	30	4.86	1.85	0.0039 *
LTA	EA	Females	17	6.71	2.11	14	5.59	1.19	0.9102 ^a^
		Males	13	6.81	2.79	16	5.29	1.83	0.6143 ^b^
		Total	30	6.75	2.39	30	5.43	1.55	0.0137 *
TA_mean_	EA	Females	17	6.50	1.72	14	5.23	1.11	0.2088 ^a^
		Males	13	7.32	1.75	16	5.07	1.97	0.7860 ^b^
		Total	30	6.85	1.75	30	5.15	1.60	0.0002 *
	AsI	Females	17	13.05	9.37	14	13.24	9.40	0.7376 ^a^
		Males	13	10.56	8.92	16	11.35	11.70	0.4667 ^b^
		Total	30	11.97	9.11	30	12.23	10.55	0.9000
RMM	EA	Females	17	5.06	2.43	14	3.81	2.26	0.7375 ^a^
		Males	13	4.82	2.21	16	3.71	1.72	0.5882 ^b^
		Total	30	4.96	2.30	30	3.75	1.96	0.0179 *
LMM	EA	Females	17	5.36	2.26	14	3.68	1.86	0.6600 ^a^
		Males	13	5.22	2.58	16	4.03	2.23	0.5742 ^b^
		Total	30	5.30	2.37	30	3.87	2.04	0.0149 *
MM_mean_	EA	Females	17	5.30	2.15	14	3.74	1.95	0.8292 ^a^
		Males	13	5.46	1.90	16	3.87	1.81	0.8558 ^b^
		Total	30	5.37	2.02	30	3.81	1.84	0.0028 *
	AsI	Females	17	13.88	9.74	14	14.75	10.70	0.7614 ^a^
		Males	13	12.79	9.54	16	14.57	13.91	0.9677 ^b^
		Total	30	13.40	9.50	30	14.65	12.31	0.6616

RTA: right anterior temporal muscle, LTA: left anterior temporal muscle, TA: anterior temporal muscles, RMM: right masseter muscle, LTA: left masseter muscle, MM: masseter muscles, EA: electrical activity (μV/μV%), AsI: Asymmetry Index (%), SD: standard deviation, ^a^ *p* value for differences in EA/AsI between females and males in the myofascial pain group, ^b^ *p* value for differences in EA/AsI between females and males in the control group, * statistically significant difference.

**Table 3:** EMG activity of the masticatory muscles during MVC in the myofascial pain and control groups.

Region	Variable	Gender	Myofascial Pain Group			Control Group			*p*-Value
			*n*	Mean	SD	*n*	Mean	SD	
RTA	EA	Females	17	114.55	28.80	14	140.46	51.17	0.1547 ^a^
		Males	13	98.01	33.06	16	134.32	59.67	0.7663 ^b^
		Total	30	107.38	31.29	30	137.19	55.00	0.0125 *
LTA	EA	Females	17	113.27	34.76	14	126.92	49.65	0.9025 ^a^
		Males	13	114.92	37.71	16	133.71	50.06	0.7127 ^b^
		Total	30	113.98	35.44	30	130.54	49.13	0.1399
TA_mean_	EA	Females	17	113.91	28.06	14	133.69	34.59	0.4025 ^a^
		Males	13	106.46	33.71	16	134.01	53.54	0.5742 ^b^
		Total	30	110.68	30.31	30	133.86	44.93	0.0797
	AsI	Females	17	11.27	7.80	14	14.41	19.09	0.2954 ^a^
		Males	13	8.48	9.67	16	6.36	9.63	0.0702 ^b^
		Total	30	10.06	8.62	30	10.12	15.10	0.2771
RMM	EA	Females	17	103.08	35.99	14	128.38	32.81	0.7764 ^a^
		Males	13	106.86	35.47	16	118.05	51.68	0.5258 ^b^
		Total	30	104.72	35.20	30	122.87	43.49	0.0807
LMM	EA	Females	17	105.16	41.09	14	117.40	33.94	0.7439 ^a^
		Males	13	100.17	41.02	16	131.26	49.23	0.3841 ^b^
		Total	30	103.00	40.42	30	124.79	42.66	0.0468 *
MM_mean_	EA	Females	17	104.12	36.33	14	122.89	28.12	0.9639 ^a^
		Males	13	103.51	35.60	16	124.66	49.56	0.9073 ^b^
		Total	30	103.86	35.40	30	123.83	40.32	0.0460 *
	AsI	Females	17	9.67	8.96	14	10.99	10.85	0.9371 ^a^
		Males	13	9.95	10.45	16	6.84	8.37	0.2471 ^b^
		Total	30	9.79	9.46	30	8.78	9.67	0.6822

RTA: right anterior temporal muscle, LTA: left anterior temporal muscle, TA: anterior temporal muscles, RMM: right masseter muscle, LTA: left masseter muscle, MM: masseter muscles, EA: electrical activity (μV/μV%), AsI: Asymmetry Index (%), SD: standard deviation, ^a^ *p* value for differences in EA/AsI between females and males in the myofascial pain group, ^b^ *p* value for differences in EA/AsI between females and males in the control group, * statistically significant difference.

**Table 4:** Data of the area under ROC curve, best cut-off value, sensitivity, and specificity of sEMG in identifying children with myofascial pain and non-TMD subjects at rest.

Region	Variable	AUC (95% CI)	SE	*p*-Value	Cut-Off Value	TP	FP	FN	TN	Se (%)	Sp (%)
RTA	EA	0.681 (0.546–0.816)	0.069	0.0160 *	4.50	27	17	3	13	90.0	43.3
LTA	EA	0.644 (0.504–0.785)	0.072	0.0546	7.85	11	2	19	28	36.7	93.3
TA_mean_	EA	0.738 (0.614–0.862)	0.063	0.0016 *	5.75	20	10	10	20	66.7	66.7
	AsI	0.509 (0.361–0.658)	0.076	0.9000	2.83	27	23	3	7	90.0	23.3
RMM	EA	0.689 (0.541–0.815)	0.070	0.0180 *	3.95	19	9	11	21	63.3	70.0
LMM	EA	0.711 (0.577–0.844)	0.068	0.0051 *	3.80	23	12	7	18	76.7	60.0
MM_mean_	EA	0.744 (0.616–0.873)	0.066	0.0011 *	3.45	26	12	4	18	86.7	60.0
	AsI	0.521 (0.366–0.675)	0.079	0.7845	6.42	26	18	4	12	86.7	40.0

RTA: right anterior temporal muscle, LTA: left anterior temporal muscle, TA: anterior temporal muscles, RMM: right masseter muscle, LTA: left masseter muscle, MM: masseter muscles, EA: electrical activity (μV/μV%), AsI: Asymmetry Index (%), AUC: area under ROC curve, SE: standard error of AUC, TP: true positive, FP: false positive, FN: false negative, TN: true negative, Se: sensitivity, Sp: specificity, * statistically significant difference.

**Table 5:** Data of the area under ROC curve, best cut-off value, sensitivity, and specificity of sEMG in identifying children with myofascial pain and non-TMD subjects during MVC.

Region	Variable	AUC (95% CI)	SE	*p*-Value	Cut-Off Value	TP	FP	FN	TN	Se (%)	Sp (%)
RTA	EA	0.667 (0.529–0.805)	0.070	0.0266 *	145.5	28	18	2	12	93.3	40.0
LTA	EA	0.559 (0.412–0.706)	0.075	0.4333	165.9	28	22	2	8	93.3	26.7
TA_mean_	EA	0.632 (0.491–0.773)	0.072	0.0798	145.3	28	19	2	11	93.3	36.7
	AsI	0.582 (0.433–0.730)	0.076	0.2772	12.4	12	4	18	26	40.0	86.7
RMM	EA	0.624 (0.481–0.767)	0.073	0.0993	104.6	18	10	12	20	60.0	66.7
LMM	EA	0.636 (0.495–0.776)	0.072	0.0713	69.5	9	0	21	30	30.0	100.0
MM_mean_	EA	0.653 (0.514–0.792)	0.071	0.0421 *	109.7	21	12	9	18	70.0	60.0
	AsI	0.567 (0.418–0.717)	0.076	0.3711	6.5	18	10	12	20	60.0	66.7

RTA: right anterior temporal muscle, LTA: left anterior temporal muscle, TA: anterior temporal muscles, RMM: right masseter muscle, LTA: left masseter muscle, MM: masseter muscles, EA: electrical activity (μV/μV%), AsI: Asymmetry Index (%), AUC: area under ROC curve, SE: standard error of AUC, TP: true positive, FP: false positive, FN: false negative, TN: true negative, Se: sensitivity, Sp: specificity, * statistically significant difference.

## Data Availability

The datasets used to support the conclusions of this article are included within the article. Access to other data will be considered by the corresponding author upon request.
